# Prior-Cancer Diagnosis in Men with Nonmetastatic Prostate Cancer and the Risk of Prostate-Cancer-Specific and All-Cause Mortality

**DOI:** 10.1155/2014/736163

**Published:** 2014-01-30

**Authors:** Kristina Mirabeau-Beale, Ming-Hui Chen, Anthony V. D'Amico

**Affiliations:** ^1^Harvard Radiation Oncology Program, Brigham and Woman's Hospital and Department of Radiation Oncology, Dana-Farber Cancer Institute, 75 Francis Street, Boston, MA 02115, USA; ^2^Department of Statistics, University of Connecticut, Storrs, CT 06269, USA; ^3^Department of Radiation Oncology, Brigham and Women's Hospital and Dana-Farber Cancer Institute, Boston, MA 02115, USA

## Abstract

*Purpose*. We evaluated the impact a prior cancer diagnosis had on the risk of prostate-cancer-specific mortality (PCSM) and all-cause mortality (ACM) in men with PC. *Methods*. Using the SEER data registry, 166,104 men (median age: 66) diagnosed with PC between 2004 and 2007 comprised the study cohort. Competing risks and Cox regression were used to evaluate whether a prior cancer diagnosis impacted the risk of PCSM and ACM adjusting for known prognostic factors PSA level, age at and year of diagnosis, race, and whether PC treatment was curative, noncurative, or active surveillance (AS)/watchful waiting (WW). *Results*. At a median followup of 2.75 years, 12,453 men died: 3,809 (30.6%) from PC. Men with a prior cancer were followed longer, had GS 8 to 10 PC more often, and underwent WW/AS more frequently (*P* < 0.001). Despite these differences that should increase the risk of PCSM, the adjusted risk of PCSM was significantly decreased (AHR: 0.66 (95% CI: (0.45, 0.97); *P* = 0.033), while the risk of ACM was increased (AHR: 2.92 (95% CI: 2.64, 3.23); *P* < 0.001) in men with a prior cancer suggesting that competing risks accounted for the reduction in the risk of PCSM. *Conclusion*. An assessment of the impact that a prior cancer has on life expectancy is needed at the time of PC diagnosis to determine whether curative treatment for unfavorable-risk PC versus AS is appropriate.

## 1. Introduction

While favorable-risk (PSA ≤ 20; T2b category or less; Gleason score ≤ 7 [1]) prostate cancer (PC) can have a long natural history [2] and is often curable, unfavorable-risk PC (which comprises approximately 20% of cases) accounts for the majority of prostate cancer deaths [3]. Men of PC bearing age are also at risk for a metachronous cancer (i.e., history of or subsequent diagnosis of another cancer). When considering life expectancy in men with PC, competing risks are particularly relevant in men with favorable-risk disease [4–10], in order to avoid overtreatment of PC where the potential toxicities of treatment can be sustained with no prolongation in survival.

To our knowledge, no study has investigated the impact that the comorbidity of a prior cancer has on the risk of PCSM.

Therefore, we used a SEER population database registry to evaluate the impact that a prior cancer had on the risk of PCSM and all-cause mortality (ACM) in men with newly diagnosed, node negative, nonmetastatic PC, adjusting for age at and year of diagnosis, race, initial treatment (curative or noncurative) or active surveillance (AS) or watchful waiting (WW), and known PC prognostic factors.

## 2. Methods

### 2.1. Patients Selection and SEER Data Registry

We used a population-based registry, SEER [11], in order to identify 166,104 men diagnosed with prostate cancer between 2004 and 2007. The inclusion period was limited to 2004–2007 when PSA data was available. The registries report information on age, date of diagnosis, demographics, tumor characteristics, surgical treatment, radiation therapy, overall survival, and cancer specific survival [11].

### 2.2. Followup and Determination of the Cause of Death

The cut-off date for determination of death was December 31, 2008. A standard decision algorithm that uses International Classification of Diseases was used to process causes of death from death certificates [12]. This study was determined to be exempted from review by the Institutional Review Board of the Dana-Farber Cancer Institute/Brigham and Women's Hospital.

### 2.3. Statistical Methods

#### 2.3.1. Comparison of the Distribution of Clinical Characteristics for the Study Cohort Stratified by Whether a History of Cancer Existed at the Time of the PC Diagnosis or Not

We compared the distribution of clinical characteristics of the 166,104 men amongst those with a cancer diagnosis preceding their PC diagnosis versus those without a prior cancer. The Wilcoxon rank-sum test [13] was used to compare the distribution of the continuous clinical characteristics and a Mantel-Haenszel chi square test [14] was used to compare the distribution of categorical covariates.

#### 2.3.2. Risk of PCSM and ACM

We used a Fine and Gray [12] competing risks and Cox regression [15] to assess whether there was an association between risk of PCSM and ACM, respectively, in men diagnosed with a prior cancer versus no prior cancer adjusting for known PC prognostic factors, curative, noncurative treatment or AS/WW, race, and age at and year of diagnosis of PC. Time zero was defined as the date of the PC diagnosis.

Adjusted (*A*) and unadjusted hazard ratios (HR) were calculated and are reported along with their 95% confidence intervals (CI) and associated *P* values. A two-sided *P* value <0.05 was considered statistically significant. *R* was used for all calculations related to Fine and Gray and SAS version 9.3 (SAS Institute, Cary, North Carolina) was used for all remaining statistical analyses.

#### 2.3.3. Estimates of PCSM and ACM

The cumulative incidence method [16] was used to calculate estimates of PCSM stratified by whether the patient had a history of cancer or not at the time of his PC diagnosis. Age adjusted comparisons of the estimates were performed using Fine and Gray's regression. ACM was defined as one minus overall survival. Estimates of ACM were calculated using the method of Kaplan and Meier [17] and age adjusted estimates [18] stratified by whether the patient had a history of cancer or not at the time of his PC diagnosis were compared using an age adjusted log rank *P* value.

## 3. Results 

### 3.1. Comparison of the Distribution of Clinical Characteristics for the Study Cohort Stratified by Whether a History of Cancer Existed at the Time of the PC Diagnosis or Not

After a median followup of 2.75 years, 12,453 men died: 3,809 (30.6%) from PC. Prior to the diagnosis of PC, 1,457 malignancies occurred at a median of 4.8 years. As shown in Table 1, men with a prior cancer were significantly older at diagnosis (median age: 72 versus 66 years, *P* = 0.001), were followed longer (median follow up: 3.0 versus 2.75 years, *P* < 0.001), were more likely to have high-risk PC (30.1% versus 26.8%, *P* = 0.01) based on the occurrence of Gleason 8 to 10 PC (19.2% versus 15.1%, *P* < 0.001), and underwent WW or AS more frequently (30.5% versus 22.5%, *P* < 0.001). In addition, they were also less likely to be African American (7.4% versus 12.3%, *P* < 0.001) and they were more likely to have clinical tumor stage T1c (42.0 versus 37.5%, *P* < 0.001) and were more likely to be diagnosed earlier in time (*P* = 0.035).

### 3.2. Risk of PCSM and ACM

As shown in Table 2, for men with a history of cancer at the time of the PC diagnosis versus not having the risk of PCSM was significantly decreased (AHR: 0.66 (95% CI: (0.45, 0.97); *P* = 0.033), whereas the risk of ACM was significantly increased (AHR: 2.92 (95% CI: 2.64, 3.23); *P* < 0.001) as shown in Table 3. Given the differences shown in Table 1 that should have led to an increase in the risk of PCSM amongst men with versus without a prior cancer, these results suggest competing risks and non curative PC treatment accounted for the observed reduction in the risk of PCSM.

### 3.3. Estimates of PCSM and ACM Stratified by Prior History of Cancer

**Figure 1:**
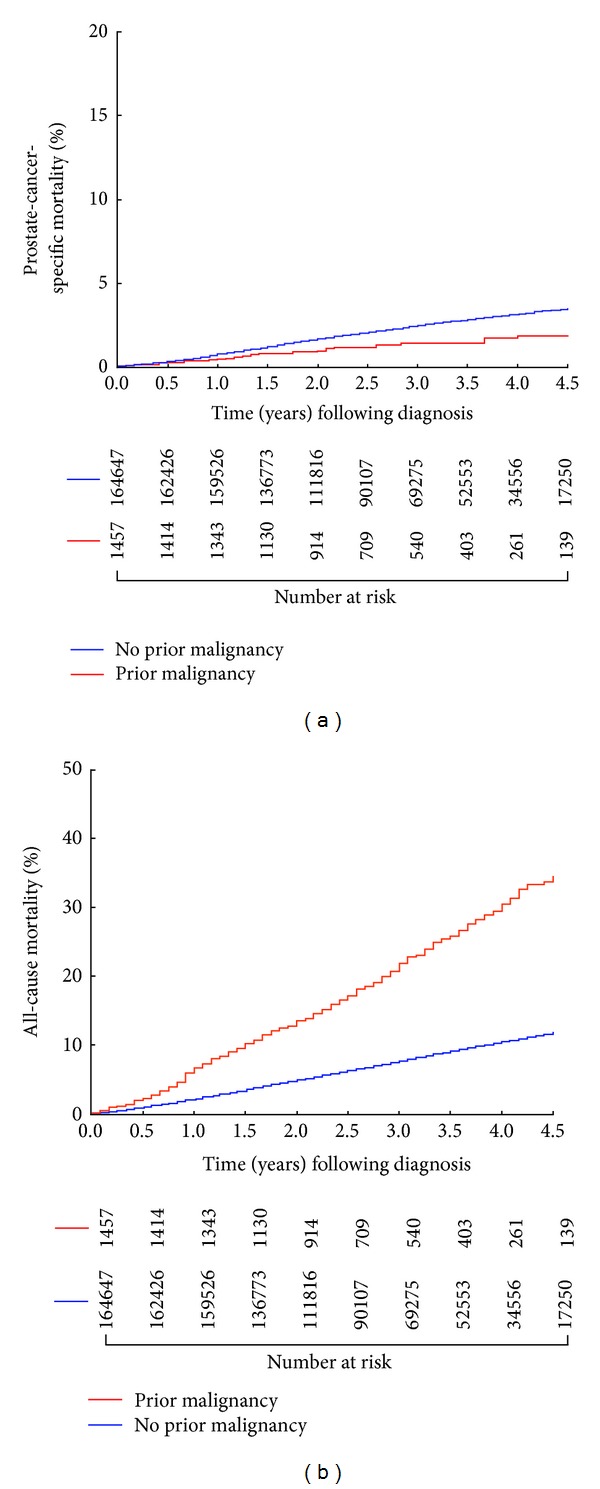
(a) Age adjusted cumulative incidence estimates of prostate-cancer-specific mortality following prostate cancer diagnosis in men with or without a history of a prior malignancy *P* = 0.012. (b) Age adjusted estimates of overall survival or all-cause mortality estimates following prostate cancer diagnosis in men with or without a history of a prior malignancy *P* < 0.001.

As shown in Figure 1(a), the age-adjusted cumulative incidence of PCSM was significantly lower for men with a prior cancer as compared to those without a history of cancer prior to the PC diagnosis (*P* = 0.012) However, age adjusted estimates of all-cause mortality were significantly higher (*P* < 0.001) for men with a prior cancer as compared to no prior cancer, as shown in Figure 1(b).

## 4. Discussion

Overtreatment remains an issue in the United States for men with low-risk PC; a target population for whom greater use of AS may be more appropriate particularly in men with significant comorbidity [19]. Our results indicate that men who had a history of cancer prior to the diagnosis of PC were followed longer, were older, and were more likely to have GS 8 to 10 PC and undergo WW or AS compared with those who did not have a history of cancer. However, despite the less frequent use of curative treatment in these men who had more aggressive PC that should have led to an increased risk of PCSM, these men had a significant decrease in the risk of PCSM while their risk of ACM increased significantly, suggesting that competing risks (prior cancer and other comorbidities) and not curative PC treatment accounted for the reduction of PCSM. The clinical significance of these findings is that AS should be more judiciously employed in men with competing risks. Specifically, while men with low risk PC are offered AS if their life expectancy is less than 10 years as per the 2013 NCCN guidelines [20] for men with unfavorable-risk PC (i.e., intermediate or high-risk) where AS would not be the preferred strategy by the existing NCCN guidelines, those who have had a prior cancer diagnosis should be considered for AS if the expected rate of cure of the prior CA is low.

Several points require further discussion. First, a recent study has shown that men with low-risk PC are more likely to be offered AS when seen in concurrent multidisciplinary setting rather than sequentially [21]. These results suggest that when a multidisciplinary team of physicians consult on a patient AS is more likely to be recommended. Second, investigators have attempted to define novel assays such as circulating tumor cell burden [22] and gene profiling [23, 24] that can stratify patients with castration resistant PC into cohorts with longer or shorter median survivals. Such tools are needed in men with low-risk PC in order to more appropriately select men for AS.

Third, men with a prior history of cancer as shown in Table 1 were more likely to be diagnosed with Gleason score 8 to 10 prostate cancer (19.2% versus 15.1%; *P* < 0.001). This likely reflects the fact that they were also significantly older at prostate cancer diagnosis (median age: 72 versus 66; *P* < 0.001) and advancing age has been shown to be associated with higher grade prostate cancer [25]. Also these men were more likely to be diagnosed at an earlier tumor stage (42% versus 37.5% T1c, *P* < 0.001), which may reflect more active medical monitoring with PSA screening given the history of the prior malignancy.

Potential limitations of this study include the inherent limitations associated with a SEER analysis, including limited information about the biopsy specimen (number and extent of positive cores, tertiary Gleason grade 5, perineural invasion). Also, data on comorbidity other than a prior history of cancer is lacking. Nevertheless, the results of our analysis add to the ongoing dialogue about quantifying the impact competing risks can have on life expectancy when deciding on whether curative treatment is likely to benefit a patient with favorable-risk PC. Conversely, some men who are otherwise healthy with favorable-risk PC may require immediate curative treatment of PC as opposed to AS in order to avoid PCSM. In order to ascertain who these men are randomized controlled trials evaluating curative treatment compared to AS should employ a prerandomization stratification by comorbidity using a validated metric of comorbidity [9] in order to assess whether treatment compared to AS benefits all men or only those with no or minimal comorbidity.

In conclusion, while an attempt is being made to offer men AS, the degree to which this has been occurring in the United States between 2004 and 2007 in men with a prior cancer does not appear to be adequate to avoid overtreatment. Therefore, an assessment of the impact that the prior cancer has on life expectancy is needed at the time of PC diagnosis to determine whether curative treatment for unfavorable-risk PC versus AS is appropriate.

## Figures and Tables

**Table 1:** Comparison of the distribution of clinical characteristics of the 166,104 men stratified by a history of cancer prior to the PC diagnosis versus no prior cancer.

Clinical characteristics	Number (%) of men without a prior cancer	Number (%) of men with a prior cancer	*P* value
	*N* = 164,647	*N* = 1,457	
Race			
African American	20208 (12.3%)	108 (7.4%)	<0.001
Other	144439 (87.7%)	1349 (92.6%)	
Year of diagnosis (range)			
2004	39609 (24.1%)	392 (26.9%)	0.035
2005	37072 (22.5%)	335 (23.0%)	
2006	42810 (26.0%)	366 (25.1%)	
2007	45156 (27.4%)	364 (25.0%)	
Age at diagnosis			
Median (IQR) (yr)	66 (60, 73)	72 (66, 78)	0.001
<50 yr	5206 (3.2%)	9 (0.6%)	<0.001
50–59 yr	35570 (21.6%)	115 (7.9%)	
60–69 yr	62463 (37.9%)	437 (30.0%)	
≥70 yr	61408 (37.3%)	896 (61.5%)	
Gleason score			
≤6	77736 (47.2%)	649 (44.5%)	<0.001
7	62016 (37.7%)	529 (36.3%)	
8 to 10	24895 (15.1%)	279 (19.2%)	
Clinical tumor stage			
1c	61793 (37.5%)	612 (42.0%)	<0.001
2a–2c	87327 (53.0%)	737 (50.6%)	
3-4	15527 (9.4%)	108 (7.4%)	
PSA			
Median (IQR)	6.5 (4.7, 10.6)	7.0 (4.7, 12.3)	0.59
≤4.0 ng/mL	23839 (14.5%)	230 (15.8%)	<0.001
>4.0–10.0 ng/mL	96632 (58.7%)	746 (51.2%)	
10.1–20.0 ng/mL	25360 (15.4%)	297 (20.4%)	
>20.0 ng/mL	18816 (11.4%)	184 (12.6%)	
Risk group			
Low risk	28682 (17.4%)	240 (16.5%)	0.01
Intermediate risk	91819 (55.8%)	778 (53.4%)	
High risk	44146 (26.8%)	439 (30.1%)	
Treatment approach			
Curative	123674 (75.1%)	954 (65.5%)	<0.001
Other Rx	4017 (2.4%)	59 (4.1%)	
Watchful-waiting/active surveillance	36956 (22.5%)	444 (30.5%)	
Median followup (IQR) (years)	2.75 (1.85, 3.92)	3.0 (2.0, 4.17)	<0.001

CI: confidence interval. Dx: diagnosis. IQR: interquartile range. PC: prostate cancer. Yr: year.

**Table 2:** Unadjusted and adjusted hazard ratios for prostate-cancer-specific mortality for patient and treatment factors.

Patient and treatment factors	Number of men	Number of events	Univariate analysis (HR with 95% CI)		Multivariate analysis (AHR with 95% CI)	
			Unadjusted HR	*P* value	Adjusted HR	*P* value
Age at diagnosis in years	166104	3809	1.07 (1.066, 1.075)	<0.001	1.01 (1.005, 1.012)	<0.001
PSA level in ng/mL at diagnosis (per log unit increase)	166104	3809	3.68 (3.56, 3.81)	<0.001	2.02 (1.93, 2.11)	<0.001
Gleason score						
<6	78385	334	1 (ref.)	—	1 (ref.)	—
7	62545	834	3.24 (2.86, 3.68)	<0.001	2.58 (2.26, 2.94)	<0.001
8–10	25174	2641	26.65 (23.78, 29.86)	<0.001	9.46 (8.29, 10.79)	<0.001
Tumor stage at diagnosis						
1c	62405	1341	1 (ref.)	—	1 (ref.)	—
2a–2c	88064	1584	0.82 (0.76, 0.88)	<0.001	0.96 (0.89, 1.04)	0.34
3-4	15635	884	2.66 (2.44, 2.89)	<0.001	1.56 (1.42, 1.72)	<0.001
Race						
African American	20316	624	1.42 (1.30, 1.55)	<0.001	1.15 (1.05, 1.26)	0.002
Other	145788	3185	1 (ref.)	—	1 (ref.)	—
Year of diagnosis in years	166104	3809	0.93 (0.90, 0.96)	<0.001	0.92 (0.89, 0.95)	<0.001
Management approach						
Curative	124628	1142	1 (ref.)	—	1 (ref.)	—
Other Rx	4076	454	12.5 (11.25, 13.98)	<0.001	4.88 (4.27, 5.59)	<0.001
Watchful-waiting/active surveillance	37400	2213	6.72 (6.25, 7.22)	<0.001	3.23 (2.94, 3.54)	<0.001
History of prior cancer at time of PC diagnosis						
No	164647	3779	1 (ref.)	—	1 (ref.)	—
Yes	1457	30	0.86 (0.60, 1.23)	0.41	0.66 (0.45, 0.97)	0.033

HR: hazard ratio. CI: confidence interval. Dx: diagnosis. HR: hazard ratio. PC: prostate cancer. Ref: reference. Rx: treatment.

**Table 3:** Unadjusted and adjusted hazard ratios for all-cause mortality for patient and treatment factors.

Patient and treatment factors	Number of men	Number of events	Univariate analysis (HR with 95% CI)		Multivariate analysis (AHR with 95% CI)	
			Unadjusted HR	*P* value	Adjusted HR	*P* value
Age at diagnosis in years	166104	12453	1.09 (1.089, 1.093)	<0.001	1.05 (1.046, 1.051)	<0.001
PSA level in ng/mL at diagnosis (per log unit increase)	166104	12453	2.09 (2.06, 2.13)	<0.001	1.38 (1.36, 1.41)	<0.001
Gleason score						
<6	78385	3571	1 (ref)	—	1 (ref)	—
7	62545	4048	1.49 (1.42, 1.56)	<0.001	1.35 (1.29, 1.42)	<0.001
8–10	25174	4834	4.76 (4.56, 4.97)	<0.001	2.46 (2.34, 2.58)	<0.001
Tumor stage at diagnosis						
1c	62405	5198	1 (ref.)	—	1 (ref.)	—
2a–2c	88064	5726	0.75 (0.73, 0.78)	<0.001	0.98 (0.94, 1.02)	0.28
3-4	15635	1529	1.17 (1.11, 1.24)	<0.001	1.30 (1.22, 1.38)	<0.001
Race						
African American	20316	1950	1.36 (1.30, 1.43)	<0.001	1.45 (1.38, 1.52)	<0.001
Other	145788	10503	1 (ref.)	—	1 (ref.)	—
Year of diagnosis in years	166104	12453	0.94 (0.92, 0.96)	<0.001	0.96 (0.94, 0.98)	<0.001
Management approach						
Curative	124628	5077	1 (ref.)	—	1 (ref.)	—
Other Rx	4076	1023	6.90 (6.45, 7.37)	<0.001	3.50 (3.25, 3.75)	<0.001
Watchful-waiting/active surveillance	37400	6353	4.62 (4.46, 4.80)	<0.001	2.61 (2.50, 2.72)	<0.001
History of prior cancer at time of PC diagnosis						
No	164647	12050	1 (ref.)	—	1 (ref.)	—
Yes	1457	403	4.08 (3.69, 4.50)	<0.001	2.92 (2.64, 3.23)	<0.001

HR: Hazard ratio. CI: Confidence Interval. Dx: diagnosis. HR: Hazard ratio. PC: prostate cancer. Ref: reference. Rx: treatment.
